# The Increase of *Lactobacillus* Species in the Gut Flora of Newborn Broiler Chicks and Ducks Is Associated with Weight Gain

**DOI:** 10.1371/journal.pone.0010463

**Published:** 2010-05-04

**Authors:** Emmanouil Angelakis, Didier Raoult

**Affiliations:** Unité des Rickettsies, CNRS UMR 6020, IFR 48, Faculté de Médecine, Université de la Méditerranée, Marseille, France; Charité-Universitätsmedizin Berlin, Germany

## Abstract

**Background:**

A bacterial role in the obesity pandemic has been suspected based on the ingestion of probiotics that can modify the gut flora. The objective of our study was to determine if increased *Lactobacillus* sp. in the gut flora of newborn broiler chicks and ducks could result in weight gain increase.

**Methodology:**

Female broiler chicks (*Gallus gallus domesticus*) and ducks (*Anas platyrhynchos domestica*) were separated into one control and two experimental groups, and inoculated once or twice with 4×10^10^ *Lactobacillus* spp. per animal in PBS, or with PBS alone. Fecal samples were collected before and at 24 hours, 2, 4, 8, 16 and 30 days after the inoculation. DNA was extracted from the stools, and qPCR assays were performed on a MX3000™ system for the detection and quantification of *Lactobacillus* sp., *Bacteroidetes* and *Firmicutes*, using a quantification plasmid. Animals were measured and sacrificed 60 days after the beginning of the experiment, and livers were collected and measured.

**Principal Findings:**

Chicks inoculated once and twice with *Lactobacillus* weighed 10.2% (*p* = 0.0162) and 13.5% (*p* = 0.0064) more than the control group animals, respectively. Similarly, ducks inoculated once and twice weighed 7.7% (*p* = 0.05) and 14% (*p* = 0.035) more than those in the control group, respectively. Liver mass was also significantly higher in inoculated animals compared to the control group. Inoculation with *Lactobacillus* sp. increased the DNA copies of *Lactobacillus* spp. and *Firmicutes* in the stools. *Bacteroidetes* remained stable, and only the second *Lactobacillus* sp. inoculation significantly decreased its population in chicks. The ratio of DNA copies of *Firmicutes* to those of *Bacteroidetes* increased to as much as 6,4 in chicks and 8,3 in ducks.

**Conclusions:**

Differences in the intestinal microbiota may precede weight increase, as we found that an increase of *Lactobacillus* sp. in newborn ducks and chicks preceded the development of weight gain.

## Introduction

The manipulation of the gut microbiota through the administration of probiotics and antibiotics has been used for growth promotion in farm animals for 50 years and is regulated by the Food and Drug Administration (FDA) in the United States [1] and by the European Commission in Europe [2]. Microorganisms used in animal food in the European Union (EU) are mainly strains of gram-positive bacteria belonging to the *Bacillus*, *Enterococcus*, *Lactobacillus*, *Pediococcus*, *Streptococcus* species and strains of yeast belonging to the *Saccharomyces cerevisiae* and *Kluyveromyces* species [2]. The manipulation of the gut microbiota by growth promoters has had a large impact on the livestock and poultry industries [3]. Probiotics were initially used to prevent episodic diarrhea in poultry, as they reduce the intestinal colonization by *Salmonella* [4] and *Clostridium perfringens* [5]. However, it was found that they promote weight gain even in the absence of diarrheal outbreaks [6].

Recently, we and others hypothesized that bacteria may play a role in the obesity pandemic due to the ingestion of probiotics that modify the gut flora [7], [8], and we stressed the necessity for further investigation of the effects of routinely adding bacteria to food [9]. Recently, type 2 diabetes mellitus was associated with compositional changes in the intestinal microbiota, as diabetics presented a significantly lower proportion of *Firmicutes* and a higher proportion of *Bacteroidetes* and *Proteobacteria* [10]. Obese diabetic subjects also presented significantly higher levels of *Lactobacillus* sp. [10]. In another study, obese patients presented significantly higher concentrations of *Lactobacillus* sp. in their feces than lean controls [11]. Moreover, we found that treatment with vancomycin in humans resulted in major and significant weight gain [12]. We speculated that the weight gain was induced by the growth-promoting effect of *Lactobacillus* sp. in patients who had been treated by vancomycin, as these bacteria are known to be resistant to glycopeptides [12]. Functional foods, such as yogurts and cheese, that are commonly consumed by adults and children contain the same *Lactobacillus* sp. in about the same concentrations as used for decades to promote growth in agriculture [13], [14]. In our study, we intragastrically administered a single dose of *Lactobacillus* sp. in broiler chicks and found that this inoculation was associated with significant weight gain [6]. The objective of our study was to determine whether the increase of *Lactobacillus* sp. in the gut flora of newborn broiler chicks could result in weight gain and to test the effects of such an inoculation on ducks.

## Results

Animals had the same weight prior to the experiment, as there were no significant differences between the experimental and the control groups for chicks and ducks (*p*>0.05) (Table 1).

**Table 1:** Animals' body weight and liver mass at the beginning and at the end of the experiment.

Body weight	Day	Control gr±SD	1 inoculation gr±SD	*P*	2 inoculations gr±SD	*p*
Broiler chicks	0	94.2±6.7	86.5±11	0.65	88.5±8	0.71
	60	1623±145	1809±185	0.0162	1878±255	0.0064
Ducks	0	82.8±16	85.7±10	0.61	85.2±11.7	0.86
	60	2472±357	2679±266	0.05	2876±468	0.035
Liver mass						
Broiler chicks		47.7±8.7	60.3±6	0.026	63±5.8	0.011
Ducks		79.6±16	110.6±26	0.0068	120.3±36	0.0054

### Chicks

Chicks inoculated with *Lactobacillus* spp. showed a faster increase in body weight compared to the control animals. On day 60, the body weight of the control animals (mean gram ± SD) was 1623±145, whereas the body weight of the animals inoculated once and twice was 1809±185 (*p* = 0.0162) and 1878±255 (*p* = 0.0064), respectively. On day 60, the liver weight of the control group animals (mean gram ± SD) was 47.7±8.7, whereas the liver weight of the chicks inoculated once and twice with *Lactobacillus* sp. was 60.3±6 (*p* = 0.026) and 63±5.8 (*p* = 0.011), respectively.

**Figure 1:**
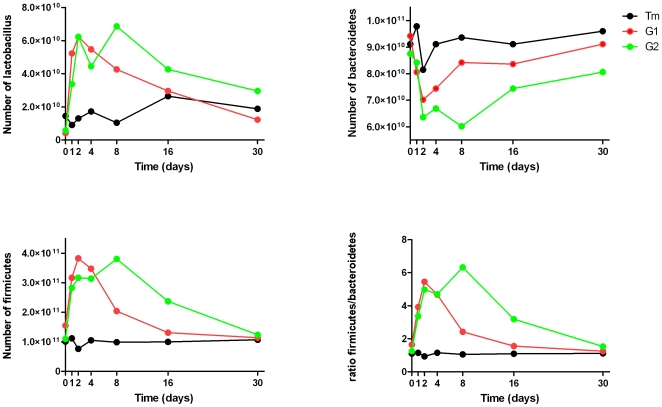
Changes in the population of *Lactobacillus* spp, *Firmicutes* and *Bacteroidetes* between the control and the experimental groups in chicks. Results were based on the mean number of DNA copies of a quantification plasmid [31]. Tm, control group; G1, chicks inoculated once with *Lactobacillus* spp.; G2, chicks inoculated twice with *Lactobacillus* spp.

Before the inoculation there was no difference between the experimental and the control groups with respect to the mean number of DNA copies of *Lactobacillus* spp., *Firmicutes* and *Bacteroidetes*, and the numbers remained constant in the control group till day 30 (*p*<0.05) (Figure 1). On day 2, a significant difference was found in the number of DNA copies of *Lactobacillus* spp. between the control group and the two experimental groups (*p* = 0.046 and *p* = 0.041, respectively). Similarly, on day 2, the numbers of DNA copies of *Firmicutes* were significantly higher in the two experimental groups (*p* = 0.029 and *p* = 0.039, respectively) compared to the control group. Animals inoculated twice on day 8 presented significantly more DNA copies of *Lactobacillus* spp. than did the control group (*p* = 0.013) and the animals inoculated only once (*p* = 0.04). Animals inoculated twice on day 8 presented significantly more DNA copies of *Firmicutes* compared to the control group (*p* = 0.042), whereas no significant changes on day 8 were observed for DNA copies of *Firmicutes* between animals inoculated once and twice (*p* = 0.086). Between the control group and the chicks inoculated once, no changes were found in the amount of DNA copies of *Lactobacillus* spp. or *Firmicutes* after day 16 (*p* = 0.54 and *p* = 0.11, respectively). Between the control group and the chicks inoculated twice, no changes were observed in the amount of DNA copies of *Lactobacillus* spp. after day 16 (*p* = 0.3) or in the amount of *Firmicutes* after day 30 (*p* = 0.9). The mean number of DNA copies of *Bacteroidetes* was not significantly different between animals in the control group and animals inoculated once during the 30 days of the experiment. Between the control group and animals inoculated twice, a significant difference in the number of DNA copies on day 8 was only found for *Bacteroidetes* (*p* = 0.047).

The ratio of the mean number of DNA copies of *Firmicutes* to those of *Bacteroidetes* in the control group remained constant during the 30 days of the experiment. However, in the experimental groups, *Lactobacillus* spp. inoculation increased this ratio. In chicks inoculated once, the largest difference between *Firmicutes* and *Bacteroidetes* with respect to the amount of DNA copies was observed on day 2, in which the *Firmicutes*/*Bacteroidetes* ratio was 5.49-fold greater than that in the control group. After day 8, the *Firmicutes*/*Bacteroidetes* ratio was similar between chicks inoculated once and the control group. We found the largest difference between *Firmicutes* and *Bacteroidetes* in animals inoculated twice on day 8, as the *Firmicutes*/*Bacteroidetes* ratio was 6.4-fold greater than that in the control group. On day 30, no difference was observed in the *Firmicutes*/*Bacteroidetes* ratio between animals inoculated twice and the control group.

### Ducks

*Lactobacillus* spp. inoculation had the same growth-promoting effects in ducks as it did in chicks. On day 60, the body weight (mean gram ± SD) of control animals was 2472±357, whereas the body weight of ducks inoculated once and twice with *Lactobacillus* spp. was 2679±266 (*p* = 0.05) and 2876±468 (*p* = 0.035), respectively. The liver weight on day 60 of the control group was 79.6±16, whereas that of ducks inoculated once and twice with *Lactobacillus* spp. was 110.6±26 (*p* = 0.0068) and 120.3±36 (*p* = 0.0054), respectively.

**Figure 2:**
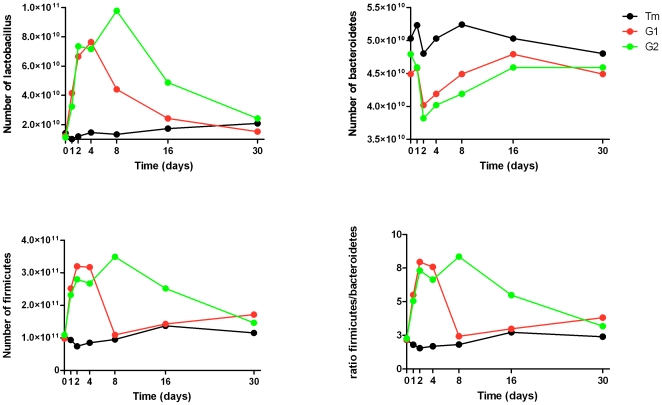
Changes in the population of *Lactobacillus* spp, *Firmicutes* and *Bacteroidetes* between the control and the experimental groups in ducks. Results were based on the mean number of DNA copies of a quantification plasmid [31]. Tm, control group; G1, ducks inoculated once with *Lactobacillus* spp.; G2, ducks inoculated twice with *Lactobacillus* spp.

qPCR revealed that before the inoculation, there was no difference between the experimental and control groups in the mean numbers of DNA copies of *Lactobacillus* spp., *Firmicutes* and *Bacteroidetes*, and the numbers remained constant in the control group during the 30 days of the experiment (Figure 2). A significant difference was observed in the number of DNA copies of *Lactobacillus* spp. on day 2 after inoculation between the control group (1.22×10^10^ DNA copies of *Lactobacillus* spp.) and the two experimental groups (*p* = 0.032 and *p* = 0.02, respectively). The mean number of DNA copies of *Firmicutes* on day 2 was significantly different between the control and the two experimental groups (*p* = 0.03 and *p* = 0.04, respectively). Ducks inoculated twice on day 8 displayed significantly more DNA copies of *Lactobacillus* spp. than did the control group (*p* = 0.01) and chicks inoculated once (*p* = 0.05). At that time point, the amount of DNA copies of *Firmicutes* were also significantly different between ducks inoculated twice and those inoculated once (*p* = 0.04) and between ducks inoculated twice and the control group (*p* = 0.02). Between the control group and the ducks inoculated once, no changes were observed in the amount of DNA copies of *Lactobacillus* and *Firmicutes* after day 8 (*p* = 0.08 and *p* = 0.7, respectively). Between the control group and the ducks inoculated twice, no changes were found in the amount of DNA copies of *Lactobacillus* and *Firmicutes* on day 30 (*p* = 0.08 and *p* = 0.7, respectively). The mean number of DNA copies of *Bacteroidetes* was not significantly different between animals in the control group and animals inoculated once (largest difference on day 4, *p* = 0.098) or twice (largest difference on day 8, *p* = 0.065).

The *Firmicutes*/*Bacteroidetes* ratio remained constant in the control group during the 30 days of the experiment. Although no significant changes were observed in the population of *Bacteroidetes*, the ratio of DNA copies for *Firmicutes*/*Bacteroidetes* increased in the experimental groups. The greatest difference between *Firmicutes* and *Bacteroidetes* with respect to DNA copies for ducks inoculated once was observed on day 2, when the number of *Firmicutes* was 7.9-fold higher than that of *Bacteroidete*s. In ducks inoculated twice, the highest ratio was observed on day 8, when the *Firmicutes*/*Bacteroidetes* ratio was 8.3. No difference was found in the *Firmicutes*/*Bacteroidetes* ratio after day 8 or day 30 between the control group and ducks inoculated once, or between the control group and ducks inoculated twice, respectively.

## Discussion

Using this experimental model, we found that even one dose of *Lactobacillus* spp. in newborn chicks accelerated weight gain and resulted in significant differences in body weight. Weight gain and differences in body weight were greater when a second dose of *Lactobacillus* spp. was administered. The chicks inoculated with *Lactobacillus* spp. displayed a significant increase not only in their body weight but also in their liver mass. Our results confirmed and extended the previous study of Khan *et al*. [6], who inoculated a single dose of either *L. fermentum* or of a strain of *Lactobacillus* sp. named Autruche 4 in 1-week-old female broiler chicks. Inoculation with the *Lactobacillus* spp. led to significantly greater weight gain and liver mass on day 29 [6]. In the present study, we found that *Lactobacillus* spp. inoculation presented the same growth-promoting effects on body weight and liver mass in ducks. In an independent study (unpublished) in collaboration with INRA (Institut National de la Recherche Agronomique) in ducks with free food access inoculated with the same *Lactobacillus* spp. (4×10^10^ bacteria/animal) we did not find evidence of weight gain although inoculated ducks presented a significant increase in liver mass.

*Lactobacillus* spp. probiotics are widely used as growth promoters in poultry, and Jin *et al.* found that the addition of 0.05%, 0.10% or 0.15% of twelve strains of *Lactobacillus* (1×10^9^ per gram) belonging to four species (*L. acidophilus, L. fermentum, L. crispatus*, and *L. brevis*) to the basal diet of 1-day-old Arbor Acres broiler chicks resulted in a significantly increased body weight compared to the control [15], [16]. The supplementation of 10^6^ CFU/gram of a transformed *L. reuteri* Pg4 strain in the food of broiler chicks from 0 to 21 days of age increased body weight and ileal villus height [17]. In another study, the daily weight gain of chickens was increased by feeding them with a diet containing a probiotic (0.1% *L. casei*) during the first 3-wk, but the average quantity of food intake was not increased [18]. In other studies, treatment with *Lactobacillus* sp. had the same growth-promoting effects as treatment with avilamycin [19], [20] and even better growth-promoting effects than chloroxytetracycline [18] or oxytetracycline [21].

We found that inoculation with *Lactobacillus* spp. in both chicks and ducks increased the population of *Firmicutes*, whereas the population of *Bacteroidetes* remained stable or slightly decreased. As a result, the *Firmicutes*/*Bacteroidetes* ratio increased after *Lactobacillus* spp. inoculation. In previous studies, a probiotic formula containing *L. reuteri*, *E. faecium*, *B. animalis*, *Pediococcus acidilactici* and *L. salivarius* displayed growth-promoting effects and significantly increased the concentrations of bacteria belonging to *Bifidobacterium* spp., *Lactobacillus* spp., and gram-positive cocci [19]. Lan *et al.* also found that 1×10^6^ *L. agilis* and *L. salivarius* enriched the diversity of *Lactobacillus* flora in the chicken jejunum and cecum by increasing the abundance and prevalence of *Lactobacillus* spp. inhabiting the intestine [22]. The same probiotic treatment, when used for 40 days in chickens, reduced the number of Enterobacteriaceae, whereas the number of lactobacilli and enterococci remained stable [23].

To the best of our knowledge, this is the first demonstration that just one *Lactobacillus* spp. inoculation early in life is capable of changing the gut flora and result in a weight increase. Analysis of the gut flora in genetically obese (leptin deficient ob/ob) mice and obese humans showed that obesity was associated with a reduction in gram-negative bacteria, specifically *Bacteroidetes*, and an increase in gram-positive *Firmicutes* bacteria [8], [24]. Kalliomaki *et al.* showed that differences in the intestinal microbiota may precede the development of an overweight phenotype [25]. It was found that the number of bifidobacterial species in fecal samples during infancy was higher in children with normal weight than in children becoming overweight, who also presented a greater number of *Staphylococcus aureus* than children with a normal weight [25]. Moreover, Membrez *et al*. found that a combination of norfloxacin and ampicillin, at a dose of 1 g/L, maximally suppressed the numbers of cecal aerobic and anaerobic bacteria in *ob/ob* mice and improved fasting glycemia and oral glucose tolerance [26]. The same group identified that a 4-week antibiotic treatment with ampicillin and neomycin resulted in a reduction of *Lactobacillus* spp., *Bifidobacterium* spp., and *Bacteroides*-*Prevotella* spp. and reduced metabolic endotoxemia and the cecal content of LPS in both high-fat–fed and *ob/ob* mice [27]. Altogether, these studies support the idea that the increase of *Lactobacillus* in the gut flora is associated with weight gain [28], [29]. In our animal experiment, we found that the increase of *Lactobacillus* sp. In the intestinal microbiota preceded the development of a weight increase. However, this link remains to be established for other animal species and humans.

## Materials

After ethical approval, 30 individually weighed 4-day-old broiler chicks (female, *Gallus gallus domesticus*, Kabir strain) and 30 individually weighed 4-day-old ducks (female, *Anas platyrhynchos domestica*, Pekin strain) purchased from a small rural hatchery (R. Ivaldi Elevage, Font Trouvade, Saint Maximin La Sainte, Baume, France) were randomly allocated to one control group and two experimental groups (10 animals/group). Animal procedures were conducted according to local regulations of animal welfare. The light/dark schedule was 14 hours of light and 10 hours of darkness; the room temperature was maintained at 22±2°C and the humidity at 55±5%.

Animals in the experimental groups were inoculated on day 0 according to a previously described method with *Lactobacillus* sp. (4×10^10^ bacteria/animal) originally isolated from an ostrich, suspended in 1 mL of phosphate-buffered saline (PBS) (pH 7.0) [6]. This *Lactobacillus* sp., is closely related (i.e., 96% similarity in 16S rRNA gene sequence) to *L. fermentum* (CIP 102980). For the second experimental group, a second inoculation with the same *Lactobacillus* dose (4×10^10^ bacteria/animal) was repeated on day 7. Animals in the control group were inoculated with PBS alone. The food quantity was the same for all groups. Fecal samples were collected from the anus by the use of a swab before the *Lactobacillus* sp. inoculation, and at 24 hours as well as 2, 4, 8, 16 and 30 days after inoculation. Animals were sacrificed 60 days after the beginning of the experiment, and livers were collected and measured.

DNA was extracted from stools using a NucleoSpin® Tissue Mini Kit (Macherey Nagel, Hoerdt, France) according to the manufacturer's instructions. Next, DNA was eluted in 100 µL of elution buffer and stored at −20°C until use. A negative control extraction of 250 µL of sterile water was introduced in each series of DNA extractions. Real-time PCR assays were performed on an MX3000™ system (Stratagene Europe, Amsterdam). The detection and quantification of *Lactobacillus* sp. were performed as described by Menard *et al*. [30]. *Bacteroidetes* and *Firmicutes* were quantified using a quantification plasmid constructed as previously described by Carcopino *et al.* [31].

For data comparison, we used EpiInfo version 6.0 software (Centers for Disease Control and Prevention, Atlanta, GA, USA). *p*<0.05 was considered as significant.
